# Periodic Fluctuation of Perceived Duration

**DOI:** 10.1177/2041669518760625

**Published:** 2018-03-08

**Authors:** Shuhei Shima, Yuki Murai, Kenichi Yuasa, Yuki Hashimoto, Yuko Yotsumoto

**Affiliations:** Department of Life Sciences, The University of Tokyo, Japan; Department of Psychology, University of California, Berkeley, CA, USA; Japan Society for the Promotion of Science, Tokyo, Japan; National Institute of Information and Communications Technology, Osaka, Japan; Graduate School of Frontier Biosciences, Osaka University, Suita, Japan; Japan Society for the Promotion of Science, Tokyo, Japan; Graduate School of Interdisciplinary Information Studies, The University of Tokyo, Japan; Japan Society for the Promotion of Science, Tokyo, Japan; Department of Life Sciences, The University of Tokyo, Japan

**Keywords:** behavioral oscillation, periodic attention, spatial attention, perception of duration, duration distortion

## Abstract

In recent years, several studies have reported that the allocation of spatial attention fluctuates periodically. This periodic attention was revealed by measuring behavioral performance as a function of cue-to-target interval in the Posner cueing paradigm. Previous studies reported behavioral oscillations using target detection tasks. Whether the influence of periodic attention extends to cognitively demanding tasks remains unclear. To assess this, we examined the effects of periodic attention on the perception of duration. In the experiment, participants performed a temporal bisection task while a cue was presented with various cue-to-target intervals. Perceived duration fluctuated rhythmically as a function of cue-to-target interval at a group level but not at an individual level when the target was presented on the same side as the attentional cue. The results indicate that the perception of duration is influenced by periodic attention. In other words, periodic attention can influence the performance of cognitively demanding tasks such as the perception of duration.

## Introduction

Our ability to discern the passage of time involves multiple mechanisms corresponding to different timescales: millisecond range, seconds-to-minutes-to-hours range, and the 24-hr light–dark cycle (Buhusi & Meck, 2005). The phenomenon of *duration distortion* has long been used to investigate mechanisms of duration perception in the millisecond range. Duration distortion refers to the phenomenon whereby perceived duration deviates from physical duration. For example, flickering stimuli (Hashimoto & Yotsumoto, 2015; Herbst, Javadi, van der Meer, & Busch, 2013), adaptation to a specific duration (Heron et al., 2012; Shima, Murai, Hashimoto, & Yotsumoto, 2016), adaptation to a specific temporal frequency (Johnston, Arnold, & Nishida, 2006), and the eccentricity of the stimulus (Kliegl & Huckauf, 2014) have all been shown to distort perceived duration; the knowledge obtained from these studies has contributed to revealing the mechanisms of time perception.

Attention is also a factor that alters perceived duration. Many studies have reported that the duration of an attended stimulus is perceived as longer than an unattended stimulus. For example, an oddball stimulus, which attracts more attention, embedded in a sequence of standard stimuli is perceived as longer than a standard stimulus (Tse, Intriligator, Rivest, & Cavanagh, 2004; Ulrich, Nitschke, & Rammsayer, 2006).

The overestimation of duration for an attended stimulus has also been reported using the Posner cueing paradigm. In a typical Posner cueing task, a brief flash called an *attentional cue* is first presented on either side of a fixation point. Then, a target stimulus is presented on the same or opposite side of the attentional cue. The participants are instructed to detect the target stimulus as fast as possible. Previous studies have shown an attentional facilitation such that the reaction time was faster when the target stimulus was presented in the same side as the attentional cue (cued location) than when the target stimulus was presented opposite of the attentional cue (uncued location; Chica, Martin-Arevalo, Botta, & Lupianez, 2014; Posner & Cohen, 1984). Studies using the Posner cueing paradigm to measure time perception require participants to judge the duration of the target stimulus. These studies have shown that the target stimulus presented on the cued location is perceived longer (Enns, Brehaut, & Shore, 1997; Mattes & Ulrich, 1998; Seifried & Ulrich, 2011; Yeshurun & Marom, 2008; but see Chen & O’Neill, 2001).

These attention-induced distortions of perceived duration can be explained not only by the distortion of the duration itself but also by the misperception of the stimulus onset or offset. In fact, attention is known to alter the temporal resolution of vision (Hein, Rolke, & Ulrich, 2006; Rolke, Dinkelbach, Hein, & Ulrich, 2008). For example, the onset of the attended stimulus can be perceived earlier (Enns et al., 1997; Rolke, Ulrich, & Bausenhart, 2006; Shore, Spence, & Klein, 2001), while the offset of the attended stimulus can be perceived later (Rolke et al., 2006). However, it should be noted that the perceived onset and offset timing alone do not determine the perceived duration. For example, the interval between the perceived onset and the perceived offset does not necessarily match the perceived duration (Bendixen, Grimm, & Schröger, 2006). Furthermore, duration distortion can be observed without changes in the perceived timing of the onset or offset (Johnston et al., 2006; Kaneko & Murakami, 2009).

In a classical Posner cueing paradigm, a shortening of the interval between the attentional cue and the target onset (cue-to-target interval) leads to a faster reaction time in the cued versus uncued location (attentional facilitation). Conversely, when the cue-to-target interval is long, the reaction time is slower in the cued versus uncued location (inhibition of return [IOR]; Posner & Cohen, 1984). Recently, periodic fluctuations in behavioral performance have been reported using densely sampled cue-to-target intervals in the Posner cueing paradigm (Fiebelkorn, Saalmann, & Kastner, 2013; Landau & Fries, 2012; Song, Meng, Chen, Zhou, & Luo, 2014). This periodic fluctuation, called behavioral oscillation, is different from the classical time course of attention (attentional facilitation and IOR) in that the behavioral oscillation has higher frequencies of performance fluctuation. It has been suggested that these behavioral oscillations reflect the periodic mechanism of attention (VanRullen, Carlson, & Cavanagh, 2007; VanRullen & Dubois, 2011). Studies of behavioral oscillation report performance fluctuations at the theta frequencies (Fiebelkorn et al., 2013; Hickok, Farahbod, & Saberi, 2015; Landau & Fries, 2012; Tomassini, Spinelli, Jacono, Sandini, & Morrone, 2015) or at the alpha frequencies (de Graaf et al., 2013; Romei, Gross, & Thut, 2012; Spaak, de Lange, & Jensen, 2014). The behavioral oscillation occurs in antiphase between the cued and uncued locations (Landau & Fries, 2012), suggesting that once the attentional cue attracts attention to the cued location, attention is alternately directed to cued and uncued locations.

The purpose of this study was twofold. The first aim was to investigate the periodic mechanism of attention. Most studies of behavioral oscillation employ a simple target detection task with brief target stimuli. However, it is unknown if the behavioral oscillation observed in that task can also be observed in cognitively demanding tasks with longer target stimuli. Such differences in task and target duration are known to alter the effect of attention. For example, IOR occurs earlier in detection tasks than in discrimination tasks (Klein, 2000; Lupiáñez, Milán, Tornay, Madrid, & Tudela, 1997). Also, a longer target duration induces stronger attentional facilitation than brief target duration (Martín-Arévalo, Chica, & Lupiáñez, 2013; Maruff, Yucel, Danckert, Stuart, & Currie, 1999). In this study, we investigated whether the periodic mechanism of attention influences performance on the duration judgment task, which is cognitively demanding with a longer target durations. If periodic attention influences the perception of duration, the perceived duration of a target stimulus presented after the attentional cue should fluctuate rhythmically as a function of the cue-to-target interval.

The second aim of our study was to investigate the role of periodic attention on duration perception. As mentioned previously, attention distorts perceived duration by distorting perceived duration itself or by misperception of the onset or offset of a stimulus. In this study, we investigated whether the perception of duration was influenced by periodic attention to the timing of the stimulus onset. The cue-to-target interval was defined as the interval between the attentional cue and the target onset. The cue-to-target interval was sampled densely to carefully manipulate attention to target onset timing.

## Method

### Participants

Ten students from the University of Tokyo (one author and nine naive participants, eight males and two females, two left-handed and eight right-handed individuals, 21.2 ± 1.2 years of age) participated in this experiment. All participants had normal or corrected-to-normal vision. All participants gave written informed consent to participate in the experiment in accordance with the Declaration of Helsinki. The protocol was approved by the institutional review boards of the University of Tokyo, and all experiments were carried out in accordance with the guidelines set by the Ethics Committee of the University of Tokyo.

### Apparatus

Stimuli were generated using Matlab [v. 2012b], Psychtoolbox [v. 3.0.11], Palamedes toolbox [v. 1.8.0] (Brainard, 1997; Pelli, 1997). Stimuli were presented on a gamma-corrected CRT monitor (DiamondtronM2 RDF223H, Mitsubishi, Tokyo, Japan) controlled by iMac OS X 10.9.5 (Apple, 1024 × 768 pixels, 120 Hz refresh rate). The experiment was conducted in a dark room. The viewing distance was 57.3 cm, and participants were asked to stabilize their head on a chin rest.

### Stimuli

**Figure 1.**
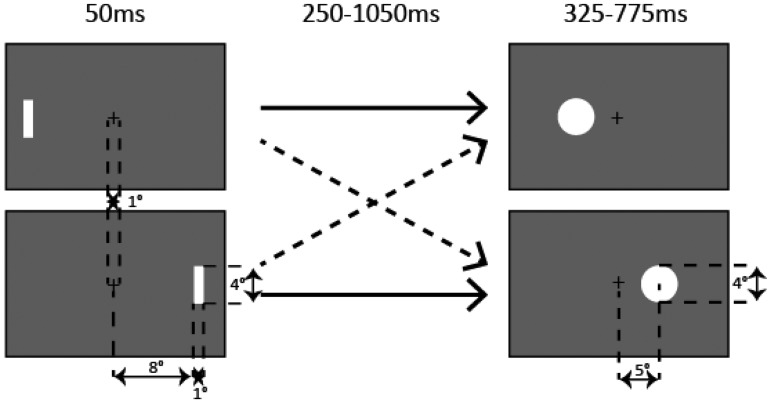
The stimuli used in the experiment. Left: The attentional cue was briefly presented on either side of the fixation point. Right: Following the cue and cue-to-target interval, a target stimulus was presented on either side of the fixation point. In the cued condition, the attentional cue and the target stimulus were presented on the same side (solid lines). In the uncued condition, they were presented on the opposite side (dashed lines).

The stimuli used in this experiment are shown in Figure 1. The standard and target stimuli were white disks (92 cd/m^2^, 4° in diameter) presented on either side of the fixation point (black cross, 1° × 1°) on a gray background (23 cd/m^2^, 40° × 30°). The distance between the fixation point and the center of the disk was 5°. The fixation point was presented at the center of the screen throughout the sessions. The attentional cue was a white rectangle (92 cd/m^2^, 4° × 1°) briefly presented (50 ms) on either side of the fixation point. The distance between the center of the fixation point and the closer side of the rectangle was 8°.

### Procedure

Participants performed a temporal bisection task which was composed of a learning phase and a testing phase. In the learning phase, a short standard stimulus (325 ms) and a long standard stimulus (775 ms) were randomly presented on either side of the fixation point at the center of the screen. Participants were asked to learn these two standard durations and indicate whether the presented stimulus was short or long by pressing a button. After the learning phase, a testing phase started. The attentional cue was briefly presented on either side of the fixation point. Following the cue, a target stimulus was presented on either side of the fixation point. The duration of the target stimulus was chosen randomly from seven durations (325–775 ms, 75-ms step), and the cue-to-target interval (interval between the onset of the attentional cue and the onset of the target stimulus) was randomly chosen from 33 durations (250–1,050 ms, 25-ms step). In the cued condition, the target stimulus was presented on the same side as the attentional cue (cued location). In the uncued condition, it was presented on the opposite side of the attentional cue (uncued location). Participants were asked to judge which of the two standard durations from the learning phase most closely matched the duration of the target stimulus. The intertrial interval (interval between the response and the onset of the attentional cue in the next trial) was randomly jittered in the range of 700 to 1,700 ms.

Participants registered their responses by pressing an up-arrow key or a down-arrow key with the forefinger of their dominant hand. The key assignments were counterbalanced across participants. They were also instructed to fixate on the fixation point throughout the sessions and to not count the durations (Rattat & Droit-Volet, 2012).

The number of trials in the testing phase was 9,240, and these trials were divided into 60 blocks across 10 days, resulting in 154 trials per block and 6 blocks per day. Half of the trials were cued conditions, and the other half were uncued conditions. The cued trials and the uncued trials were randomly presented. The number of trials per target duration per cue-to-target interval was 20. To learn and retain the internal representation of the standard durations, learning phases were embedded in the testing phase. Each block was separated into four parts, and learning phases were inserted at the beginning of each part: 12 learning trials were inserted before the first trial of each block, and 4 learning trials were inserted before 40th, 78th, and 117th trials of each block.

On the first day of the experiment, participants underwent a practice block with 140 trials to accustom themselves to the temporal bisection task before the main experiment. Thus, the number of blocks on the first day was seven: one practice block and six experimental blocks. The procedure was the same as the main experiment except that the attentional cue was not presented before the target stimulus.

### Data Analysis

To improve the signal-to-noise ratio, we performed a binning procedure used in Fiebelkorn et al. (2013). For example, trials from the 250 -, 275 -, and 300-ms cue-to-target intervals were merged per target duration, and these merged trials were considered as trials from the 275-ms cue-to-target interval. This 50-ms bin was shifted forward by 25 ms, and trials from the 275 -, 300 -, and 325-ms cue-to-target intervals were merged per target duration and considered as trials from the 300-ms cue-to-target interval. This procedure was repeated throughout the duration of the cue-to-target interval (250–1,050 ms). As a result, cue-to-target intervals decreased from 33 (250–1,050 ms) to 31 (275–1,025 ms). Before binning, the number of trials per cue-to-target interval was 20 per one target duration. After binning, the number of trials per cue-to-target interval increased to 60 per one target duration.

In each cue-to-target interval, the proportions of *long* responses were calculated for each target duration, plotted as a function of target duration, and then fit with a cumulative normal distribution function:

$$y=\frac{\beta }{\sqrt{2\pi }}\int_{-\infty }^{x}\text{exp}(-\frac{\beta^{2}(x-a{)}^{2}}{2})$$

- Next, the parameters α and β were estimated. Fitting and estimation were conducted using Palamedes toolbox for Matlab (Prins & Kingdom, 2009). The parameter α, which indicates the duration that gives 50% long responses, was determined as the bisection point at a particular cue-to-target interval and indicated perceived duration. The bisection points were calculated for each cue-to-target interval and calculated separately for cued and uncued conditions.

After estimating the bisection points, we tested whether the bisection points of the cued and uncued conditions fluctuated rhythmically. The fluctuation of bisection points may contain both the slower temporal component reflecting the classical time course of attention (attentional facilitation and IOR) and the faster temporal component reflecting periodic attention. We first removed this slower component by detrending with a first-order linear function and filtered by a third-order Butterworth filter (cutoff frequency: 3 Hz; see Fiebelkorn et al., 2013; Song et al., 2014). A single-frequency sine function was then fit to this high-passed fluctuation of bisection points in each condition and in each participant with the least squares method. The best fit frequency of the sine function was estimated. The coefficient of determination (*R*^2^) of this fitting procedure was used as an index of periodicity in the bisection points (de Graaf et al., 2013; Fiebelkorn et al., 2011; Picazio et al., 2014):

individualR2=1-∑(yi-y^i)2∑(yi-y¯i)2groupR2=∑individualR2n

where *y*_i_ is the bisection point at the *i*th cue-to-target interval, y^i is the value of the fit sine function at the *i*th cue-to-target interval, $\bar{y}$ is the mean of the bisection points across all cue-to-target intervals, and *n* is the number of participants. Note that the group *R*^2^ value was the simple mean of the individual *R*^2^ values, thus the fitting of psychometric functions, detrending, and the fitting of sine functions were always performed at an individual level. The individual *R*^2^ and group *R*^2^ show the extent to which the single-frequency sine function fits the fluctuation of bisection points at an individual level and at a group level, respectively (Fiebelkorn et al., 2011). In other words, a significantly high *R*^2^ value indicates rhythmic fluctuation of the bisection points.

Permutation testing was conducted to test whether the *R*^2^ value was statistically significant. The proportion of long responses was permutated across cue-to-target intervals, before applying the 50-ms binning process. This permutation procedure was conducted separately for each participant and target duration. The permutated data were then binned, and the *R*^2^ value was calculated for each permutation. After 2,500 iterations, a sampling distribution of *R*^2^ values was constructed. If the *R*^2^ value from the real data set was larger than the top 5% of *R*^2^ values from the sampling distribution, the *R*^2^ value was considered to be significant. We analyzed not only raw bisection points but also the difference between bisection points in the cued and uncued conditions to determine the spatial effect of attention. After calculating the difference in bisection points’ between the two conditions, the same procedure (fitting to a single-frequency sine function, *R*^2^ value calculation, and permutation testing) was conducted.

Landau and Fries (2012) reported that the effect of periodic attention is stronger when the attentional cue is presented on the right hemifield. To confirm this attentional benefit, the same analysis was conducted only using trials in which the attentional cue was presented on the right hemifield. In addition, we investigated whether discrimination accuracy fluctuated rhythmically. The slope of the psychometric function (i.e., the parameter β) was regarded as an indicator of discrimination accuracy. The same procedure was conducted to the slope parameters in the cued and the uncued conditions, and the difference in slopes between the cued and the uncued conditions.

In addition to the analysis of periodicity, the classical time course of attention, such as attentional facilitation and IOR, was also examined via a two-way repeated measures analysis of covariance. The null hypothesis was that there would be no bisection point difference between the cued and uncued conditions and no linear trend of the bisection point sequences. The factors were 2 conditions (cued and uncued) × 31 cue-to-target intervals.

## Results

**Figure 2.**
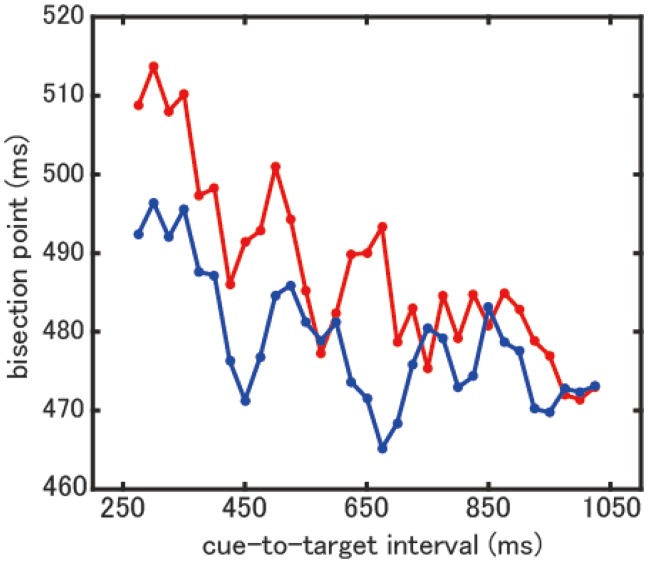
The bisection points as a function of the cue-to-target interval in a typical participant. The red lines show the cued condition and the blue lines show the uncued condition.

**Figure 3.**
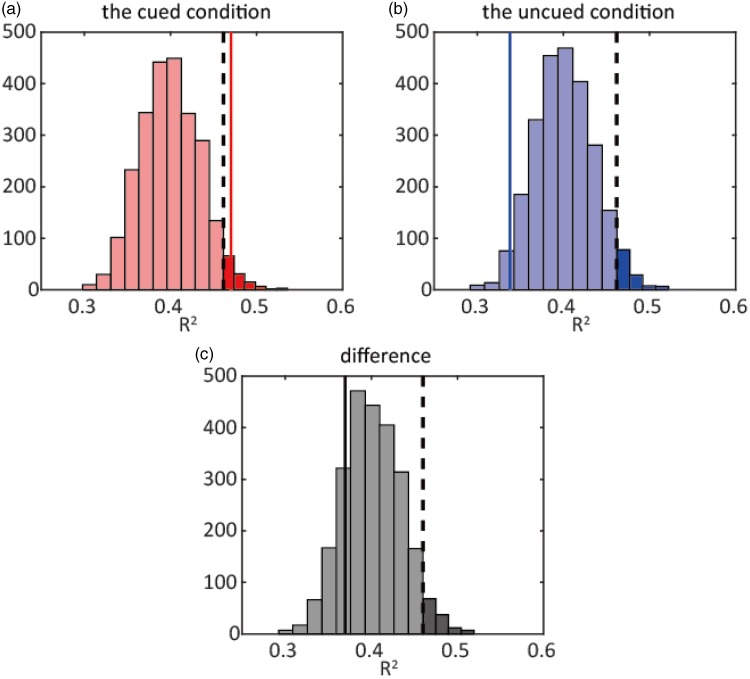
Sampling distribution of group *R*^2^ values after permutation. Solid lines show the group *R*^2^ values from the real data set and dashed lines show the top 5%. (a) The cued condition, *p* = .029, (b) the uncued condition, *p* > .05, and (c) the difference between bisection points, *p* > .05.

Psychometric functions in a typical participant are shown in Supplementary Figure 1. The fluctuation of bisection points in the cued and the uncued conditions in a typical participant is shown in Figure 2 and in all participants in Supplementary Figure 2. After detrending with a first-order linear function, filtered by a third-order Butterworth filter, and fitting to a single-frequency sine function, the *R*^2^ value was calculated. The fit results for all participants in the cued and the uncued conditions are shown in Supplementary Figure 3. Group-level permutation testing showed that the group *R*^2^ value for the observed data was significantly higher than that of the permutated data in the cued condition (*p* = .029) but not in the uncued condition nor in the difference between bisection points (*p* > .05; Figure 3). This result indicates that single-frequency sine functions fit well with the fluctuation of bisection points in the cued condition at a group level. In other words, the bisection points fluctuated rhythmically as a function of the cue-to-target interval. In addition, to determine whether such rhythmic fluctuations were observed at an individual level, individual permutation testing was conducted using individual *R*^2^ values. *R*^2^ values were not significant in almost all participants (*p* > .05) except one participant in the cued condition (*p* = .01) and one participant in the difference between bisection points (*p* = .05). These results indicate that single-frequency sine functions did not fit well with the fluctuation of the bisection points at an individual level. Therefore, the rhythmic fluctuation of the bisection points could not be observed at an individual level. The frequencies of the sine function, *R*^2^ values, and *p* values calculated for both group and individual assessments are shown in Supplementary Table 1. In the cued condition, the frequencies of the best fit sine functions were around the theta band (4.9 ± 1.5 Hz (*SD*)). In addition, the group *R*^2^ values were calculated with the same procedure using trials where the attentional cue was presented in the right hemifield only. No significant difference was found in any conditions (*p* > .05). Also, we did not observe significant periodic fluctuations of the slope parameters in any conditions (*p* > .05).

**Figure 4.**
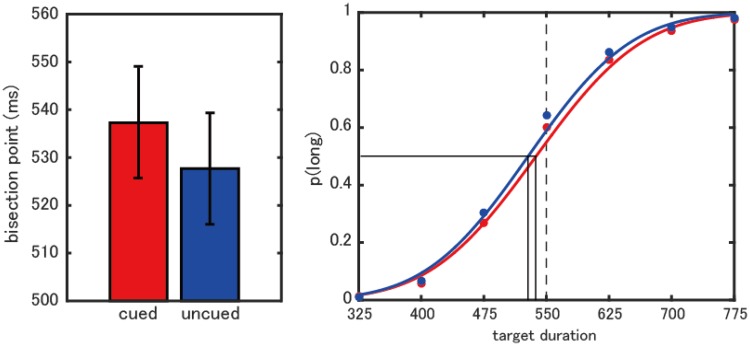
Averaged data across all cue-to-target intervals. Left: The averaged bisection points. Right: The averaged psychometric functions across all cue-to-target intervals and all participants. Red shows the cued condition and blue shows the uncued condition. Solid black lines indicate bisection points and dashed black line indicates intermediate target duration.

The bisection points between the cued and the uncued conditions were also compared (Figure 4). A two-way repeated measures analysis of covariance revealed a significant difference in the bisection points between the cued and the uncued conditions, *F*(1,9) = 35.7, *p* < .001, but showed neither a significant trend of the bisection point as a function of the cue-to-target interval, *F*(1,9) = 2.0, *p* = .19, nor an interaction, *F*(1,9) < 1, *p* = .66. These results indicate that the bisection point was larger in the cued condition than in the uncued condition.

## Discussion

### Perceived Duration Fluctuated Rhythmically as a Function of Cue-to-Target Interval

In this study, we investigated whether the perception of duration is influenced by periodic attention. It was found that perceived duration fluctuated rhythmically as a function of cue-to-target interval in the cued condition at a group level but not at an individual level. Periodic fluctuations in behavioral performance have been reported using rather simple target detection tasks for briefly presented target stimuli. Although previous research on spatial attention has pointed out that longer target duration and difficult tasks alter, or sometimes negate, the effect of attention (Klein, 2000; Martín-Arévalo et al., 2013; Maruff et al., 1999), here we suggested the possibility that the periodic mechanism of attention also influences the performance of a cognitively demanding task such as the duration perception task.

In the experiment, attention to the timing of the target onset was manipulated by densely sampled cue-to-target intervals. We will introduce two explanations for the periodic fluctuation of perceived duration. One is that attention to the timing of the target onset altered the perceived onset timing (Enns et al., 1997; Rolke et al., 2006; Shore et al., 2001). Although the perceived onset timing itself was not measured directly in our study, altered perceived onset could influence perceived duration. The other plausible explanation is that attention to the timing of the target onset altered the perceived duration itself and not via the change in the perceived onset timing. Further research is needed to dissociate these two possibilities.

The results of our study can be interpreted in the context of the pacemaker–accumulator model (Gibbon, Church, & Meck, 1984; Treisman, 1963) that is one of the classical models for time perception. This model includes three components: the *pacemaker* that emits pulses to the *accumulator* and *switch* that lies between the pacemaker and accumulator. When we start measuring time, the switch is turned on and the accumulator starts to accumulate pulses from the pacemaker. When we stop measuring time, the switch is turned off, and the pulses from the pacemaker are stopped and are no longer transmitted to the accumulator. The number of accumulated pulses represents perceived duration. The attentional-gate model (Zakay & Block, 1995) suggests that the switch is influenced by attention. When attention is directed to the target stimulus, the switch can be turned on earlier and pulse transmission can start sooner. The accumulator can then accumulate more pulses that result in longer perceived duration. If periodic attention alters the switch onset timing, the number of accumulated pulses would fluctuate periodically, which would result in the periodic fluctuation of perceived duration.

Some studies have assumed that the frequency of behavioral oscillation is the same between individuals, and their analyses were conducted to fit only one frequency to the group-level mean of behavioral performance (Fiebelkorn et al., 2013; Landau & Fries, 2012). In contrast, other studies report that the frequencies of behavioral oscillations are different across individuals (de Graaf et al., 2013; Fiebelkorn et al., 2011; Romei et al., 2012). In our experiment, different frequencies were fit to each individual’s data, as in Fiebelkorn et al. (2011), to observe behavioral oscillations both when the frequencies of behavioral oscillations are the same across individuals and when the frequencies of behavioral oscillations are different across individuals. As a result, in the cued condition, we observed behavioral fluctuations at theta frequencies, which are consistent with studies that reported behavioral oscillations using target detection tasks (Fiebelkorn et al., 2013; Landau & Fries, 2012; Tomassini et al., 2015).

Significant behavioral oscillations were not detected at the individual level in our study, which may be due to the effect size of behavioral oscillation. In fact, many studies only report behavioral oscillation at a group level and not at an individual level (Drewes, Zhu, Wutz, & Melcher, 2015; Dugué, Roberts, & Carrasco, 2016; Fiebelkorn et al., 2013; Landau & Fries, 2012). A group-level analysis has greater statistical power than an individual analysis (Biau, Kernéis, & Porcher, 2008), which may lead to detection of behavioral oscillation despite a small effect size. In this study, the observed amplitude of the individual fluctuation of bisection points was small (±6 ms) relative to standard durations (325 ms and 775 ms), and standard error of each bisection point was at around ±8 ms. Such a small effect size might be the reason why behavioral oscillations were not detected at the individual level in our experiment.

We speculate that the small effect size might be a result of the experimental manipulation. For example, when the onsets of seven target durations were aligned, the offsets were not temporally aligned. This problem was inevitable because seven target durations had to be used to calculate a psychometric function for each cue-to-target interval in the temporal bisection task. There are tasks that only require one target duration such as the temporal reproduction task; however, we did not use the temporal reproduction task because it is known to have reproduction bias, especially in subsecond durations when participants reproduce the duration by pressing a button (Shi, Ganzenmüller, & Müller, 2013). Another plausible reason for the small effect size could be the large number of trials required to complete the experiments. In the experiment, participants performed six blocks per day, which took 1.5 hr each day, for 10 days. The prolonged experimental sessions seem to have induced a learning effect. Bisection points obtained in the first day were smaller than bisection points obtained in the last day, *t*(9) = 2.63, *p* = .027, while the difference in bisection points between the cued and uncued conditions was not different between the first and last days, *t*(9) = 1.33, *p* = .21. This learning effect was represented by the change in the bisection point itself, not the change in the fluctuation of bisection points, indicating that the learning effect did not influence periodic attention. However, such a long experiment might result in a practice effect that decreased the effect of attention (Lupiáñez, Weaver, Tipper, & Madrid, 2001) and led to a decrease in periodic attention. This problem was inevitable because many cue-to-target intervals had to be used to observe high-frequency behavioral oscillations. Similarly, multiple long durations had to be used in order to estimate a psychometric function.

The effect of periodic attention is known to be stronger in the right hemifield than in the left hemifield (Landau & Fries, 2012). To determine whether such an attentional benefit existed in our experiment, we conducted the same analysis using only trials in which the attentional cue was presented in the right hemifield. However, we did not find periodic fluctuations of perceived duration in either hemifield. To evaluate the hemifield-specific effect in this analysis, the number of trials had to be cut by half, which decreased the effect size. More trials will be required to examine hemifield-specific effects.

As saccadic eye movement can also affect duration perception (Morrone, Ross, & Burr, 2005), some may wonder whether behavioral oscillations might have resulted from eye movements. Several studies observed behavioral oscillations after excluding trials with eye movement, suggesting that behavioral oscillations are not completely a result of eye movement (Fiebelkorn et al., 2011, 2013; Landau & Fries, 2012; Song et al., 2014).

Several previous studies suggested that behavioral oscillation is associated with neural oscillation. For example, the frequency of behavioral oscillation correlates with individual alpha frequency in the brain (de Graaf et al., 2013; Romei et al., 2012). A study reported that behavioral oscillation derives from entrainment of neural activity in the visual cortex to external flickering stimuli (Spaak et al., 2014). Behavioral performance correlates with the phase of neural oscillation following particular timing (Busch, Dubois, & VanRullen, 2009; Busch & VanRullen, 2010; Landau, Schreyer, van Pelt, & Fries, 2015; Sherman, Kanai, Seth, & VanRullen, 2016). Future studies should investigate the neural implementation of periodic attention altering perceived duration.

Attention-induced distortion of perceived duration can be induced not only by misperception of the onset but also by misperception of the stimulus offset (Enns et al., 1997; Rolke et al., 2006; Shore et al., 2001). In this study, we showed the effect of periodic attention on the timing of target onset in the perception of duration by densely sampling intervals between the attentional cue and target onset. Further studies will be needed to discuss the effects of periodic attention on the target offset.

### Bias Induced by an Attentional Cue Might Explain Shorter Perceived Duration in the Cued Condition

Bisection points were larger in the cued condition than in the uncued condition. In other words, perceived duration was shorter in the cued condition than in the uncued condition. This result is inconsistent with studies that report an expansion of perceived duration in the cued compared to uncued condition (Enns et al., 1997; Mattes & Ulrich, 1998; Seifried & Ulrich, 2011; Yeshurun & Marom, 2008). One possibility is IOR-induced duration compression that induces shorter perceived duration in the cued condition than in the uncued condition when the cue-to-target interval is long (Osugi, Takeda, & Murakami, 2016). To test this possibility, we conducted an additional experiment with a shorter cue-to-target interval (50 ms). The detailed experimental procedure and results are provided in Supplementary Materials. We did not observe a longer perceived duration in the cued condition even in the short cue-to-target interval. This result denied the possibility of IOR-induced duration compression. The perceived duration was shorter in the cued condition independently of the cue-to-target interval.

An alternative explanation for the shorter perceived duration in the cued condition is a bias induced by an attentional cue. In the cued condition, an attentional cue was presented in the same location as the target stimulus. The short duration of the attentional cue (50 ms) might bias participants’ judgment about the duration of the subsequent target, if presented in the same location, as being shorter. Such bias has been reported in one previous study (Wiener, Thompson, & Branch Coslett, 2014).

We did not observe the typical attentional effect (i.e., duration expansion in the cued condition); thus, the typical attentional effect may be obscured by bias induced by the attentional cue. This does not necessarily deny the possibility of periodic attentional modulation; periodic attention and bias can occur simultaneously because attention-induced duration distortion is a perceptual phenomenon and bias is a cognitive phenomenon (Wiener et al., 2014). In addition, some behavioral oscillation studies reported periodic attention but not typical attentional effects (Fiebelkorn et al., 2013; Landau & Fries, 2012). They indicate that periodic attention can occur even in the absence of typical attentional effects. In fact, some studies report behavioral oscillations that do not reflect periodic attentional modulation but rather reflect periodic modulation of visual processing such as priming (Huang, Chen, & Luo, 2015) or visual categorization (Drewes et al., 2015). Therefore, the results reported in this study do not deny the possibility that the observed oscillation reflects a periodic modulation of other types of visual processing but not attention.

One candidate for a periodically modulated visual processing is duration perception. Using a priming task, Huang et al. (2015) interpreted the behavioral modulations as the periodic modulation of priming. Also, using a visual categorization task, Drewes et al. (2015) interpreted the behavioral modulations as the periodic modulation of visual categorization. By the same logic, using a duration perception task, our results can be interpreted as the periodic modulation of duration perception. If periodic mechanism exists in the duration perception, it would periodically change the perceived duration directly not via periodic changes of attention. Further study is needed to explore what was actually modulated during periodic fluctuation of the perceived duration.

### Periodic Fluctuation of Perceived Duration Was Observed Only in the Cued Condition

In this study, significant behavioral oscillation was observed only in the cued condition. This result is partially inconsistent with Landau and Fries (2012) who reported that the behavioral oscillations were in antiphase between the cued and uncued locations and concluded that multiple locations were alternately attended to. To explain this discrepancy, we need to assume multiple periodic attentional mechanisms for different locations, instead of one periodic attentional mechanism with a periodic spatial modulation. Fiebelkorn et al. (2013) reported behavioral oscillation only in the uncued condition and not in the cued condition. They also reported that the frequencies of behavioral oscillations in the uncued condition differ depending on the location relative to the cue. Their study suggested multiple periodic attentional modulations in different locations relative to the cue not one periodic attentional modulation mechanism that alternately attends to multiple locations. If different periodic attentional mechanisms separately regulate behavioral oscillations of perceived duration in the cued and uncued locations, periodic fluctuations would not necessarily be antiphase but could have different characteristics between in the cued condition and in the uncued condition.

Under this assumption, the reason of periodic fluctuation being observed only in the cued condition can be explained by the stimulus property used in our study. The effect of attention is known to decrease when the size of the attentional focus is larger because attention is allocated evenly across the broad area (Castiello & Umiltá, 1990). In a typical stimulus configuration of the Posner cueing paradigm, placeholders marked the location where the target stimulus would appear (Chica et al., 2014) and restricted the size of the attentional focus in both cued and uncued locations. In the present experiment, such placeholders indicating potential target positions were not used, in an attempt to simplify stimulus sets. Instead of placeholders, the attentional cue might work as the location cue that restricted the size of the attentional focus in the cued location, and the size of the attentional focus was restricted near the position of the attentional cue. However, the attentional cue could not restrict the size of the attentional focus in the uncued location, and therefore, the attention might be allocated evenly within the side of the screen opposite to the attentional cue. In the cued condition, the target stimulus was presented in the cued location, where the size of the attentional focus was restricted. Thus, the effect of attention might not decrease in the cued condition. On the other hand, in the uncued condition, the target stimulus was presented in the uncued location, where the size of the attentional focus was not restricted. Thus, decreased attentional effects in the uncued location could have made the behavioral oscillation in the uncued condition unobservable.

## Conclusion

We investigated whether the perception of duration is influenced by periodic attention. Perceived duration fluctuated rhythmically as a function of the cue-to-target interval only in the cued condition. This result suggested the possibility that perceived duration is distorted periodically by the periodic mechanism of attention, and that periodic attention influences performance in a cognitively demanding task such as the perception of stimulus duration.

## Supplemental Material

Supplemental material for Periodic Fluctuation of Perceived Duration Click here for additional data file. Supplemental material for Periodic Fluctuation of Perceived Duration by Shuhei Shima, Yuki Murai, Kenichi Yuasa, Yuki Hashimoto and Yuko Yotsumoto in i-Perception

## Supplementary Material

Supplementary material
