# Supplementary material for: Periodic Fluctuation of Perceived Duration
Source: Iperception. 2018 Mar 8;9(2):2041669518760625. doi: 10.1177/2041669518760625 (PMC5937633; doi:10.1177/2041669518760625)
Supplement: Supplemental material for Periodic Fluctuation of Perceived Duration [file Supplemental_material.pdf]

Periodic Fluctuation of Perceived Duration
Supplemental material

Additional Experiment: Experimental procedure and results

In the main experiment, we observed shorter perceived duration in the cued condition than in the uncued condition. It was inconsistent with the typical effect of attention on perceived duration (i.e., longer perceived duration in the cued condition). One explanation is that IOR occurred and compressed perceived duration in the cued condition. To test this possibility, an additional experiment was conducted. Five students from the University of Tokyo (one author and four naïve participants, three males and two females, all right handed individuals, 22.0 ± 1.3 years of age) participated in the additional experiment. Stimuli were presented on a gamma-corrected CRT monitor (DiamondtronM2 RDF223H, Mitsubishi) controlled by iMac OS X 10.12.2 (Apple, 800 × 600 pixels, 120 Hz refresh rate). The stimuli and procedure were the same as the main experiment, except that briefer cue-to-target intervals were used: 50 ms and 250 ms. The number of trials in the testing phase was 560 and these trials were divided into 5 blocks, resulting in 112 trials per block. The number of trials per target duration in a given cue-to-target interval was 20. Learning phases were embedded in the testing phase. Each block was separated into three parts, and learning phases were inserted at the beginning of each part: twelve learning trials were inserted before the first trial of each block, and four learning trials were inserted before the 37th and 74th trials of each block.

Supplementary Figure 4 shows the results of the additional experiment. A two-way repeated measures analysis of variance (ANOVA) was conducted on the

bisection points. Bisection points were larger in the 50 ms cue-to-target interval than in the 250 ms cue-to-target interval ($F(1,4) = 8.7$, $p = .04$). The difference in the bisection points between the cued and the uncued conditions was not significant ($F(1,4) = 3.9$, $p = .12$). There was no interaction between cue position and cue-to-target interval ($F(1,4) < 1$, $p = .76$). These results indicate that perceived duration in the cued condition was not longer than in the uncued condition, even in the shorter cue-to-target interval.

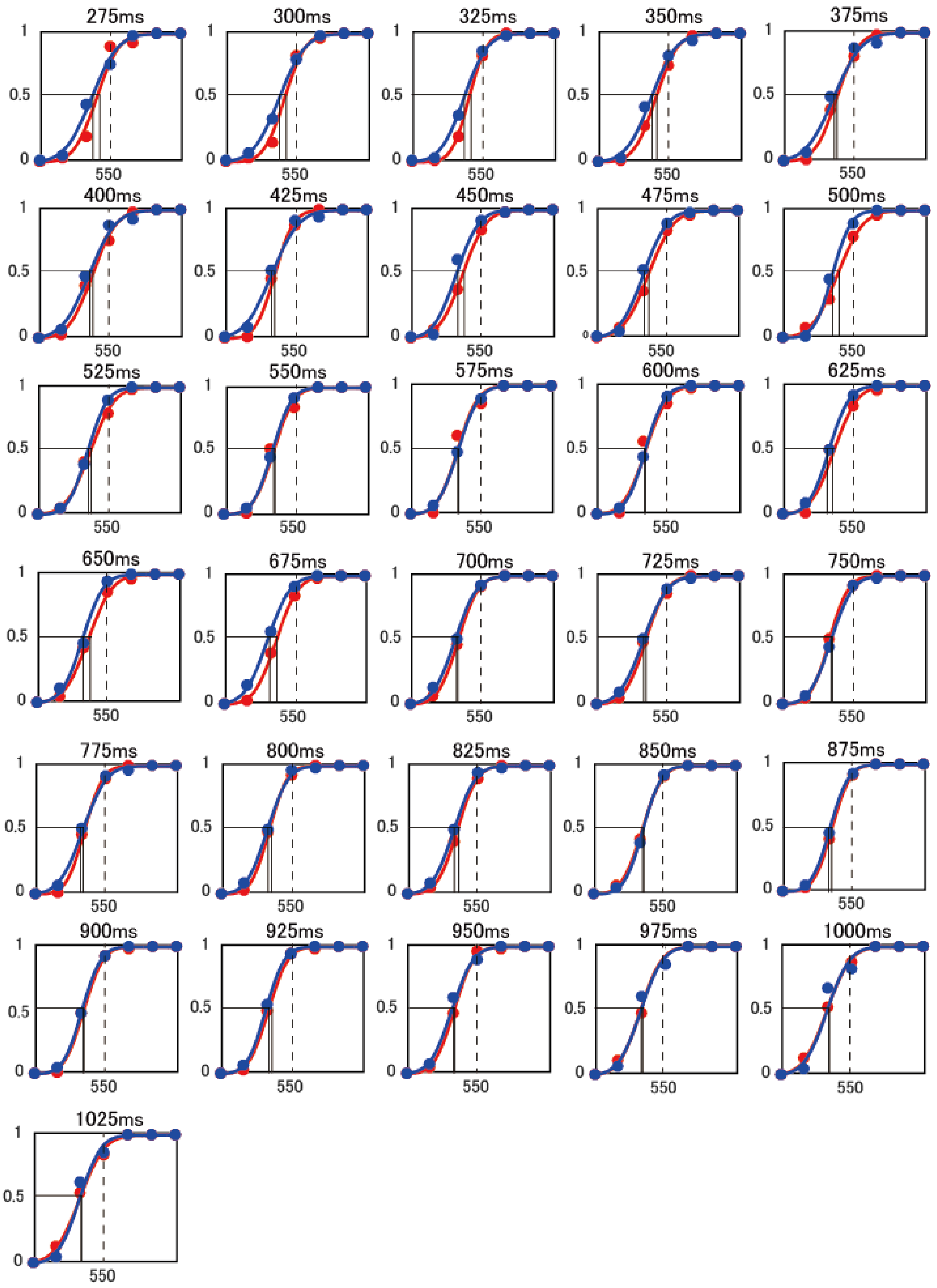
275ms
300ms
325ms
350ms
375ms
400ms
425ms
450ms
475ms
500ms
525ms
550ms
575ms
600ms
625ms
650ms
675ms
700ms
725ms
750ms
775ms
800ms
825ms
850ms
875ms
900ms
925ms
950ms
975ms
1000ms
1025ms
1
0.5
0
550

Supplementary Figure 1. Psychometric functions in each cue-to-target interval in a typical participant. The red lines show the cued condition and the blue lines show the uncued condition. Solid black lines indicate bisection points and dashed black line indicates intermediate target duration.

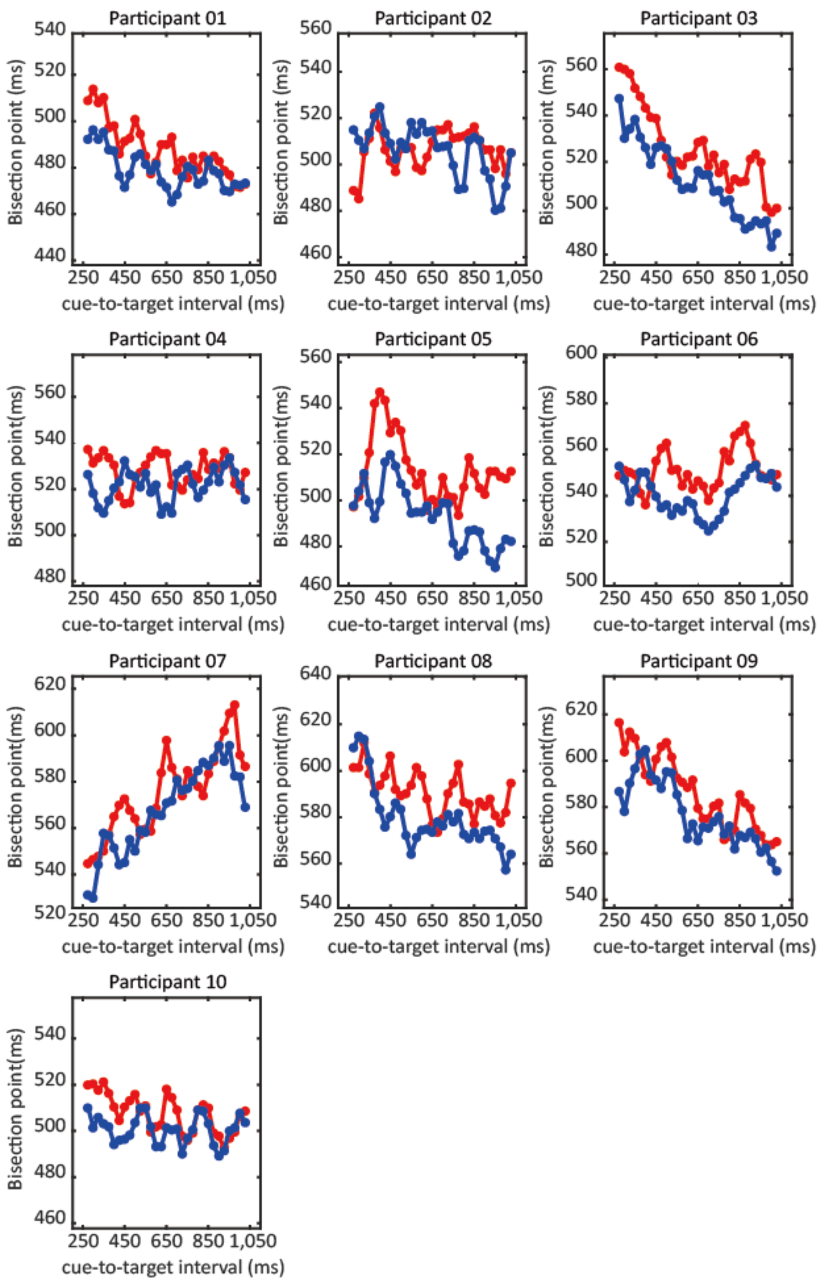

Supplementary Figure 2. The raw bisection points as a function of cue-to-target interval in each participant. The red lines show the cued condition and the blue lines show the

uncued condition.

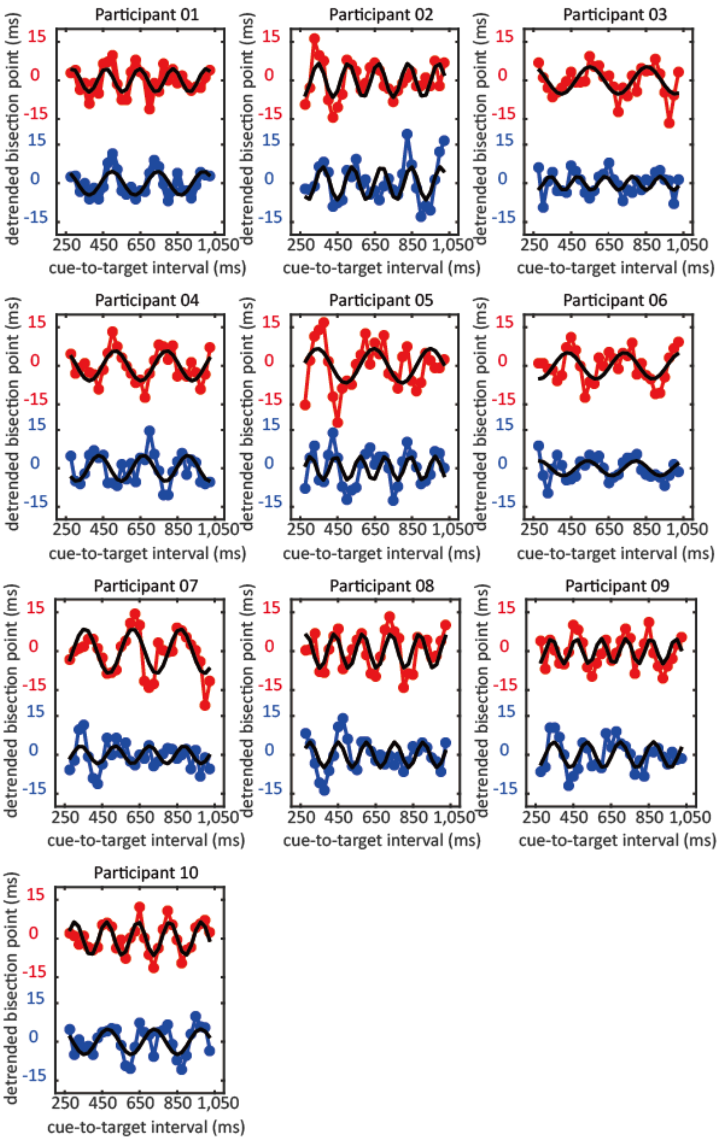

Supplementary Figure 3. The fluctuation of detrended bisection points as a function of cue-to-target interval. The red lines show the cued conditions and the blue lines show the

uncued conditions. The black lines show the best-fitted sine function in each participant.

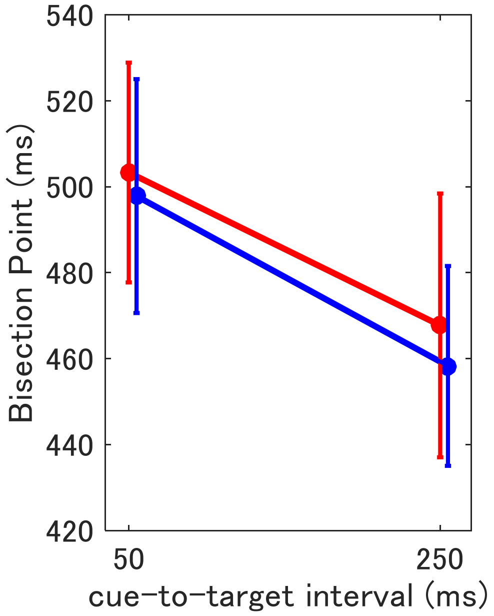

Supplementary Figure 4. The bisection points obtained in the additional experiment. The red lines show the cued condition and the blue lines show the uncued condition.

Supplementary Table 1. Frequencies of fitted sine function, R^2 values, and p-values calculated for both group and individual assessments.

Participant	Cued condition			Uncued condition			Difference		
	R^2	*p*	frequency	R^2	*p*	frequency	R^2	*p*	frequency
01	.42	.39	5.6	.47	.26	4.1	.34	.66	5.0
02	.51	.16	6.2	.41	.44	6.3	.40	.46	6.7
03	.45	.30	3.3	.21	.98	6.8	.32	.74	3.7
04	.53	.11	3.6	.41	.42	4.2	.61	.05	3.9
05	.28	.86	3.3	.26	.91	7.4	.35	.62	5.2
06	.37	.53	3.3	.25	.93	3.8	.32	.75	5.6
07	.58	.08	3.9	.23	.96	5.4	.31	.79	5.9
08	.49	.21	6.6	.35	.64	6.6	.35	.64	5.9
09	.37	.58	7.6	.38	.51	5.7	.33	.74	7.1
10	.70	.01	5.9	.40	.45	4.0	.36	.60	4.4
Group	.47	.03	4.9	.34	.97	5.5	.37	.82	5.3
